# Psychosocial Risk Factors and Cardiovascular Disease and Death in a Population-Based Cohort From 21 Low-, Middle-, and High-Income Countries

**DOI:** 10.1001/jamanetworkopen.2021.38920

**Published:** 2021-12-15

**Authors:** Ailiana Santosa, Annika Rosengren, Chinthanie Ramasundarahettige, Sumathy Rangarajan, Jephat Chifamba, Scott A. Lear, Paul Poirier, Karen E. Yeates, Rita Yusuf, Andreas Orlandini, Liu Weida, Li Sidong, Zhu Yibing, Viswanathan Mohan, Manmeet Kaur, Katarzyna Zatonska, Noorhassim Ismail, Patricio Lopez-Jaramillo, Romaina Iqbal, Lia M. Palileo-Villanueva, Afzalhusein H. Yusufali, Khalid F. AlHabib, Salim Yusuf

**Affiliations:** 1 School of Public Health and Community Medicine, Institute of Medicine, Sahlgrenska Academy, University of Gothenburg, Gothenburg, Sweden; 2 Department of Molecular and Clinical Medicine, Institute of Medicine, Sahlgrenska Academy, University of Gothenburg, and Region Västra Götaland, Sahlgrenska University Hospital, Goteborg, Sweden; 3 Population Health Research Institute, Hamilton, Ontario, Canada; 4 Department of Medicine, McMaster University and Hamilton Health Sciences, Hamilton, Canada; 5 College of Health Sciences, Department of Physiology, University of Zimbabwe, Harare, Zimbabwe; 6 Faculty of Health Sciences, Simon Fraser University c/o Healthy Heart Program, St Paul’s Hospital, Vancouver, Canada; 7 Faculté de pharmacie, Université Laval, Institut universitaire de cardiologie et de pneumologie de Québec, Québec, Canada; 8 Department of Medicine, Etherington Hall, Queen’s University, Kingston, Canada; 9 Independent University, Dhaka, Bangladesh; 10 Estudios Clínicos Latino America, Instituto Cardiovascular de Rosario, Santa Fe, Argentina; 11 Medical Research and Biometrics Center, Fuwai Hospital, National Center for Cardiovascular Diseases, Beijing, China; 12 Chinese Academy of Medical Sciences and Peking Union Medical College, Beijing, China; 13 Madras Diabetes Research Foundation and Dr, Mohan’s Diabetes Specialities Centre, Chennai, India; 14 School of Public Health, Postgraduate Institute of Medical Education and Research, Chandigarh, India; 15 Department of Social Medicine, Wroclaw Medical University, Wroclaw, Poland; 16 Department of Community Health, Faculty of Medicine, University Kebangsaan Malaysia, Kuala Lumpur, Malaysia; 17 Masira Research Institute, Universidad de Santander, Bucaramanga, Colombia; 18 Department of Community Health Sciences and Medicine, Aga Khan University, Karachi, Pakistan; 19 College of Medicine, University of the Philippines, Manila, Philippines; 20 Dubai Medical College, Hatta Hospital, Dubai Health Authority, Dubai, United Arab Emirates; 21 Department of Cardiac Sciences, King Fahad Cardiac Center, College of Medicine, King Saud University, Riyadh, Saudi Arabia

## Abstract

**Importance:**

Stress may increase the risk of cardiovascular disease (CVD). Most studies on stress and CVD have been conducted in high-income Western countries, but whether stress is associated with CVD in other settings has been less well studied.

**Objective:**

To investigate the association of a composite measure of psychosocial stress and the development of CVD events and mortality in a large prospective study involving populations from 21 high-, middle-, and low-income countries across 5 continents.

**Design, Setting, and Participants:**

This population-based cohort study used data from the Prospective Urban Rural Epidemiology study, collected between January 2003 and March 2021. Participants included individuals aged 35 to 70 years living in 21 low-, middle-, and high-income countries. Data were analyzed from April 8 to June 15, 2021.

**Exposures:**

All participants were assessed on a composite measure of psychosocial stress assessed at study entry using brief questionnaires concerning stress at work and home, major life events, and financial stress.

**Main Outcomes and Measures:**

The outcomes of interest were stroke, major coronary heart disease (CHD), CVD, and all-cause mortality.

**Results:**

A total of 118 706 participants (mean [SD] age 50.4 [9.6] years; 69 842 [58.8%] women and 48 864 [41.2%] men) without prior CVD and with complete baseline and follow-up data were included. Of these, 8699 participants (7.3%) reported high stress, 21 797 participants (18.4%) reported moderate stress, 34 958 participants (29.4%) reported low stress, and 53 252 participants (44.8%) reported no stress. High stress, compared with no stress, was more likely with younger age (mean [SD] age, 48.9 [8.9] years vs 51.1 [9.8] years), abdominal obesity (2981 participants [34.3%] vs 10 599 participants [19.9%]), current smoking (2319 participants [26.7%] vs 10 477 participants [19.7%]) and former smoking (1571 participants [18.1%] vs 3978 participants [7.5%]), alcohol use (4222 participants [48.5%] vs 13 222 participants [24.8%]), and family history of CVD (5435 participants [62.5%] vs 20 255 participants [38.0%]). During a median (IQR) follow-up of 10.2 (8.6-11.9) years, a total of 7248 deaths occurred. During the course of follow-up, there were 5934 CVD events, 4107 CHD events, and 2880 stroke events. Compared with no stress and after adjustment for age, sex, education, marital status, location, abdominal obesity, hypertension, smoking, diabetes, and family history of CVD, as the level of stress increased, there were increases in risk of death (low stress: hazard ratio [HR], 1.09 [95% CI, 1.03-1.16]; high stress: 1.17 [95% CI, 1.06-1.29]) and CHD (low stress: HR, 1.09 [95% CI, 1.01-1.18]; high stress: HR, 1.24 [95% CI, 1.08-1.42]). High stress, but not low or moderate stress, was associated with CVD (HR, 1.22 [95% CI, 1.08-1.37]) and stroke (HR, 1.30 [95% CI, 1.09-1.56]) after adjustment.

**Conclusions and Relevance:**

This cohort study found that higher psychosocial stress, measured as a composite score of self-perceived stress, life events, and financial stress, was significantly associated with mortality as well as with CVD, CHD, and stroke events.

## Key Points

**Question:**

Is there an association between psychological stress level and development of cardiovascular disease (CVD) and death?

**Findings:**

In this cohort study of 118 706 participants without prior CVD, the risk of death and congestive heart disease increased significantly as the level of stress increased, while high, but not low or moderate, stress was associated with CVD and stroke after adjustment for sociodemographic factors and health risk behaviors.

**Meaning:**

These findings emphasize the need for the development and evaluation of prevention strategies to address stress.

## Teaser

This cohort study assessed the association of a composite measure of psychosocial stress with incident cardiovascular disease and mortality in a population-based cohort from 21 low-, middle-, and high-income countries.

## Introduction

Psychological factors, including stress at work or in family life collectively, may play a role in the development of cardiovascular disease (CVD).^1^ However, stress is self-reported and could be subjective and therefore hard to measure in a standardized manner. Accordingly, the association of psychological factors with CVD risk is more controversial compared with other more traditional risk factors (eg, smoking, obesity, diabetes, high blood pressure, elevated cholesterol levels).^1,2,3,4^ Previous studies have shown an association between stress and CVD through direct or indirect pathways,^4,5,6,7^ and a 1.1- to 1.6-fold increased risk of incident coronary heart disease (CHD) and stroke associated with stress.^8,9^ A large retrospective case-control study conducted in 52 countries found that long-term stress was associated with an increased risk of acute myocardial infarction (MI) even after controlling for health behaviors.^6^ However, studies in which psychological factors are assessed retrospectively may be more prone to biases.^10^

Most studies are limited by the focus on a single conceptualization of stress and stress measurement, which may have contributed to inconsistent findings.^2,11^ Furthermore, prospective data on the association of psychological stress with the development of CVD and mortality from low-income countries and middle-income countries are scarce.^1,3,12^ Using data from the Prospective Urban Rural Epidemiology (PURE) study, a large-scale prospective cohort study from high-, middle-, and low-income countries, we evaluated the association of a composite measure of psychosocial factors with the development of CVD and all-cause mortality.

## Methods

The PURE study received ethical approval from each participating center, and all participants provided written informed consent. The protocol has been published previously online.^13^ This prospective cohort study was coordinated by the Population Health Research Institute (Hamilton, Canada), and local ethics approval was obtained by all collaborating sites. This study followed the Strengthening the Reporting of Observational Studies in Epidemiology (STROBE) reporting guideline.

### Study Design and Participants

PURE is a prospective cohort study of individuals aged 35 to 70 years at baseline in 27 countries across 5 continents, with baseline and follow-up data in 160 967 participants. We excluded 12 206 individuals with prior CVD (ie, stroke, CHD, heart failure, or other heart disease) and a further 29 784 individuals with incomplete baseline data or incomplete follow-up data (eFigure 1 in the Supplement). The enrollment of participants started in January 2001, aiming to include countries across a wide range different economic and social circumstances and varying health policies, with a proportionally larger representation from middle- and low-income countries. Countries were classified based on gross national income per capita according to the World Bank classification in 2006. Participating countries are 4 high-income countries (Canada, Saudi Arabia, Sweden, and United Arab Emirates), 12 middle-income countries (Argentina, Brazil, Chile, China, Colombia, Iran, Malaysia, Occupied Palestinian Territory, Philippines, Poland, South Africa, and Turkey) and 5 low-income countries (Bangladesh, India, Pakistan, Tanzania, and Zimbabwe). Detailed study design, sampling, enrollment and methods have been published previously.^14,15,16^ Follow-up was initiated in all sites by 2008, and we included all reported events until March 2021 in this study.

### Measures of Stress

#### Psychological Stress

Psychosocial stress was assessed by 2 single-item questions relating to stress at work and home. *Stress* was defined as feeling irritable or filled with anxiety or as having sleeping difficulties as a result of conditions at work or home. For each question, participants were asked to report to what extent they had felt stressed. In the INTERHEART study,^6^ a single-item question^17^ used in studies in Gothenburg, Sweden, since 1970 was divided into separate questions for stress at work and home. Because stress at work and home were highly correlated and because only 60% of participants were currently employed, we created a global stress scale for the purpose of this study that combined work and home stress that was graded as^1^ never experienced stress,^2^ experienced at least 1 period of stress,^3^ experienced several periods,^4^ and experienced permanent stress. Moreover, we dichotomized stress categories into participants with never stressed or at least 1 period of stress (score = 0) and several periods of stress or permanent stress (score = 1).

#### Life Events Stress

*Life events* stress was defined as whether respondents had experienced any of a number of specified major adverse life events in the past year (eg, loss of job, retirement, loss of crop or business failure, marital separation or divorce, death of spouse, death or major illness of close family member).^6^ Respondents who had experienced any major adverse life events in the last year were defined as having had life event stress.

#### Financial Stress

Level of financial stress was categorized as whether respondents had felt financial stress in the last 12 months, with the following response options: little or none, moderate, and high or severe.^6^ We regrouped these into 2 categories: little or none and moderate vs high or severe financial stress.

#### Total Stress

A composite score was calculated by summing the score of psychological stress, major life events, and financial stress. Scores ranged from 0 to 3, categorized as no stress (score = 0), low stress (score = 1), moderate stress (score = 2), and high stress (score = 3).

### Covariates

Sociodemographic characteristics included age (35-44, 45-54, or 55-70 years), sex, country income (high, medium, or low), marital status (never married, currently married or living with partner, or widowed or divorced), education (college or university, secondary or high school, or none or primary school), and location (urban or rural). Hypertension was defined as either diagnosed with hypertension and using medication or recorded systolic blood pressure 140 mm Hg or higher or diastolic blood pressure 90 mm Hg or higher. Tobacco use was defined as never, former, or current smoker. For alcohol use, a standard alcohol consumption frequency questionnaire was used (current alcohol use or not). Abdominal obesity was defined as waist circumference greater than 102 cm for men or greater than 88 cm for women. Diabetes was defined as fasting glucose measured at baseline of 126.13 mg/dL or greater (to convert to millimoles per liter, multiply by 0.0555), self-reported history of diabetes, or using a glucose-lowering agent. Family history of CVD in parents was coded as present or not present.

### Outcomes

The outcomes of interest were major CVD (a composite of cardiovascular death, stroke, MI, and heart failure), major CHD (acute MI and coronary death), stroke, and all-cause mortality. Events classified according to the *International Statistical Classification of Diseases and Related Health Problems, Tenth Revision* (*ICD-10*) are described in the eMethods in the Supplement. Information on specific events was obtained from participants or their family, who were contacted at regular intervals. Detailed information on deaths was obtained from verbal autopsies or medical records, and details on nonfatal events were obtained from hospital or physician reports using standardized definitions and review of documents. Events were adjudicated centrally in each country by trained physicians using all available documentation.

### Statistical Analysis

Direct standardization was used to calculate age- and sex-standardized incidence rates per 1000 person-years for cardiovascular events and deaths using the total PURE population as reference. The age- and sex-standardized rates were stratified by the composite score of stress. The follow-up time for each respondent was calculated from the date of baseline to a first event, death, or last follow-up.

We performed multivariable Cox frailty models with random effects to account for center clustering, which automatically adjusts for both region and country. The hazard ratios (HRs) and their 95% CIs are presented for the associations between the composite score of psychosocial stress and outcomes (all-cause mortality, major CVD, stroke, and major CHD), adjusted for age, Sex, marital status, education, country income, location and family history of CVD, hypertension, abdominal obesity, smoking, alcohol consumption, and diabetes. We also performed the analyses separately for each component of psychosocial stress (eMethods in the Supplement). In addition, we conducted a subgroup analysis to investigate potential reverse causality, where analyses were repeated dropping deaths within the first 5 years of follow-up. This analysis did not include deaths from external causes.

To study the consistency of the associations of psychosocial factors with outcomes in different geographical regions and in urban and rural settings, we performed tests for interactions for the associations between psychosocial factors and outcomes in each setting. We found no significant interactions, so the main findings are presented. We conducted sensitivity analysis using a multiple imputed data set because 19% missing data were observed for some covariates (ie, smoking status, alcohol consumption, abdominal obesity, hypertension, and psychosocial stress). We conducted multiple imputation with chained equations.^18^ Results from the sensitivity analysis showed comparable results with analyses using complete-case (eTable 1 in the Supplement). To examine whether the associations of psychosocial factors with outcomes differed between men and women, we stratified by sex. Statistical analyses and figures were generated with Stata software version 16.1 (StataCorp). *P* values were 2-sided, and statistical significance was set at *P* < .05. Data were analyzed from April 8 to June 15, 2021.

## Results

The analytic sample included 118 706 participants (mean [SD] age, 50.4 [9.6] years; 69 842 [58.8%] women and 48 864 [41.2%] men) without prior CVD and with complete baseline and follow-up data. Of these, 8699 participants (7.3%) were categorized as having high stress, 21 797 participants (18.4%) were categorized as having moderate stress, 34 958 (29.4%) were categorized as having low stress, and 53 252 participants (44.8%) were categorized as no stress. Baseline characteristics of participants are presented in Table 1. Participants who lived in high-income countries, compared with those living in low-income countries, more often reported high stress (1788 participants [11.5%] vs 979 participants [6.1%]) and less often reported no stress (4707 participants [30.2%] vs 6589 participants [40.7%]), with the same pattern for those living in urban settings compared with those living in rural settings (eg, high stress: 5086 participants [8.1%] vs 3613 participants [6.5%]). Participants who were married or living with partner and those with secondary or high school education reported no stress more often than other categories (Table 1). Participants with a high level of stress, compared with those with no stress, were younger (mean [SD] age: 48.9 [8.9] years vs 51.1 [9.8] years) and more likely to have abdominal obesity (2981 participants [34.3%] vs 10 599 participants [19.9%]), be current smokers (2319 participants [26.7%] vs 10 477 participants [19.7%]) or former smokers (1571 participants [18.1%] vs 3978 participants [7.5%]), use of alcohol (4222 participants [48.5%] vs 13 222 participants [24.8%]), and have a family history of CVD (5435 participants [62.5%] vs 20 255 participants [38.0%]) (Table 2). Baseline characteristics of the PURE respondents for each component of psychosocial factors are presented in eTables 2 through 7 in the Supplement.

**Table 1.** Baseline Socioeconomic Characteristics of the PURE Study Population by Composite Score of Psychosocial Factors

Variables	No.	Composite stress score, No. (%)				
		None (score = 0)	Low (score = 1)	Moderate (score = 2)	High (score = 3)
Country income					
High	15 588	4707 (30.2)	5360 (34.4)	3733 (23.9)	1788 (11.5)
Middle	86 937	41 956 (48.3)	24 187 (27.8)	14 862 (17.1)	5932 (6.8)
Low	16 181	6589 (40.7)	5411 (33.4)	3202 (19.8)	979 (6.1)
Education					
≤Primary school	48 117	19 375 (40.3)	14 793 (30.7)	9996 (20.8)	3953 (8.2)
Secondary or high school	45 636	23 623 (51.8)	12 597 (27.6)	6875 (15.1)	2541 (5.6)
≥College or university	24 953	10 254 (41.1)	7568 (30.3)	4926 (19.7)	2205 (8.8)
Marital status					
Never married	5534	1408 (25.4)	1799 (32.5)	1537 (27.8)	790 (14.3)
Currently married or living with partner	101 574	48 528 (47.8)	29562 (29.1)	17 223 (17.0)	6261 (6.2)
Widowed or divorced	11 598	3316 (28.6)	3597 (31.0)	3037 (26.2)	1648 (14.2)
Area					
Urban	62 725	27 359 (43.6)	18 331 (29.2)	11 949 (19.1)	5086 (8.1)
Rural	55 981	25 893 (46.3)	16 627 (29.7)	9848 (17.6)	3613 (6.5)

**Table 2.** Baseline Demographic Characteristics of the PURE Study Population by Composite Score of Psychosocial Factors

Variables	Composite stress score, No. (%)			
	None (score = 0) (n = 53 252)	Low (score = 1) (n = 34 958)	Moderate (score = 2) (n = 21 797)	High (score = 3) (n = 8699)
Age, mean (SD), y	51.1 (9.8)	50.4 (9.6)	49.7 (9.3)	48.9 (8.9)
Sex				
Men	22 620 (42.5)	14 643 (41.9)	8528 (39.1)	3073 (35.3)
Women	30 632 (57.5)	20 315 (58.1)	13 269 (60.9)	5626 (64.7)
Family history of CVD	20 255 (38.0)	17 276 (49.4)	12 310 (56.5)	5435 (62.5)
Tobacco use				
Current	10 477 (19.7)	7215 (20.6)	4931 (22.6)	2319 (26.7)
Former	3978 (7.5)	4459 (12.8)	3339 (15.3)	1571 (18.1)
Never	38 797 (72.9)	23 284 (66.6)	13 527 (62.1)	4809 (55.3)
Abdominal obesity	10 599 (19.9)	9106 (26.1)	6565 (30.1)	2981 (34.3)
Alcohol use	13 222 (24.8)	12 199 (34.9)	9182 (42.1)	4222 (48.5)
Hypertension	22 353 (42.0)	13 980 (40.0)	8590 (39.4)	3354 (38.6)
Diabetes	2314 (4.4)	1554 (4.5)	1100 (5.1)	470 (5.4)

Abbreviation: CVD, cardiovascular disease.

During a median (IQR) follow-up of 10.2 (8.6-11.9) years, there were 7428 deaths (6.3%), 5934 major CVD events, 4107 major CHD events, and 2880 stroke events. The age- and sex-standardized rates of mortality and events for the overall cohort and by groups of composite psychosocial factors are summarized in Table 3. The age- and sex-standardized event rates for all-cause deaths increased as the composite score of stress increased (eg, deaths: 7.8 [95% CI, 7.5-8.1] events per 1000 person-years in those with no stress up to 9.7 [95% CI, 8.7-10.7] events per 1000 person-years in those with high stress), as did event rates for major CHD (eg, 4.7 [95% CI, 4.5-5.0] events per 1000 person-years in those with no stress up to 5.5 [95% CI, 4.7-6.3] events per 1000 person-years in those with high stress) (Table 3). On the other hand, rates of stroke decreased from 4.3 (95% CI, 4.0-4.5) events per 1000 person-years in those with no stress to 3.2 (95% CI, 2.6-3.8) events per 1000 person-years in those with high stress.

**Table 3.** ASR per 1000 Person-Years Stratified by Composite Score of Psychosocial Factors

Outcomes	Overall (n = 118 706)		Composite stress score							
			None (score = 0) (n = 53 252)		Low (score = 1) (n = 34 958)		Moderate (score = 2) (n = 21 797)		High (score = 3) (n = 8699)	
	Events, No.	ASR (95% CI), per 1000 PY	Events, No.	ASR (95% CI), per 1000 PY^a^	Events, No.	ASR (95% CI), per 1000 PY^a^	Events, No.	ASR (95% CI), per 1000 PY^a^	Events, No.	ASR (95% CI), per 1000 PY^a^
All-cause death	7428	8.8 (8.6-9.0)	3083	7.8 (7.5-8.1)	2286	9.4 (9.0-9.8)	1506	10.4 (9.8-11.0)	553	9.7 (8.7-10.7)
CVD	5934	7.6 (7.4-7.8)	2860	7.8 (7.5-8.1)	1717	7.5 (7.1-7.9)	975	7.2 (6.7-7.7)	382	8.3 (7.3-9.2)
Coronary heart disease	4107	5.0 (4.9-5.2)	1799	4.7 (4.5-5.0)	1263	5.3 (5.0-5.6)	759	5.2 (4.8-5.7)	286	5.5 (4.7-6.3)
Stroke	2880	3.7 (3.6-3.9)	1568	4.3 (4.0-4.5)	763	3.4 (3.1-3.6)	394	3.0 (2.7-3.3)	155	3.2 (2.6-3.8)

Abbreviations: ASR, age- and sex-standardized rate; CVD, cardiovascular disease; PY, person-years.

^a^ For standardization, total PURE population was used as the reference population.

The Figure shows multiple-adjusted HRs for the outcomes of interest (ie, all-cause death, major cardiovascular disease, major CHD, and stroke). After adjustment for socioeconomic and demographic factors, smoking, physical activity, alcohol consumption, hypertension, diabetes, country income, and family history of CVD, the associations with risk of death, CVD, and CHD were attenuated but still significant. The risks increased with increasing the level of stress for death (low stress: HR, 1.09 [95% CI, 1.03-1.16]; high stress: HR, 1.17 [95% CI, 1.06-1.29]) and for CHD (low stress: HR, 1.09 [95% CI, 1.01-1.18]; high stress: HR, 1.24 [95% CI, 1.08-1.42]). High stress, but not low or moderate levels of stress, was significantly associated with CVD (HR, 1.22 [95% CI, 1.08-1.37]) and stroke (HR, 1.30 [95% CI, 1.09-1.56]) after adjustment.

**Figure.**
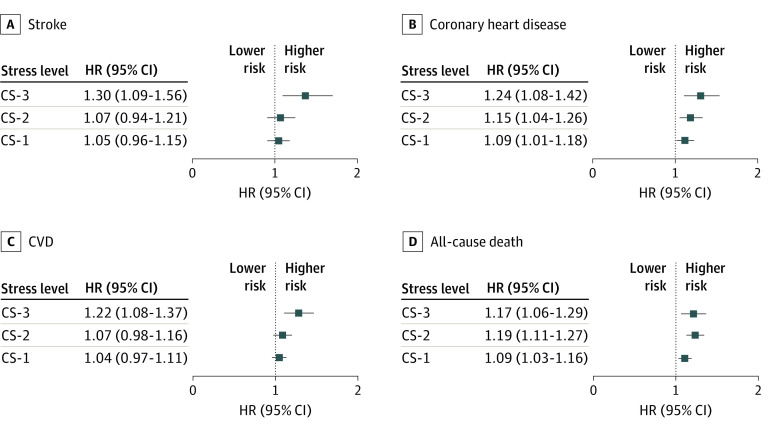
Adjusted Hazard Ratios (HRs) for All-Cause Mortality, Cardiovascular Disease (CVD), Coronary Heart Disease, and Stroke by Composite Score (CS) of Psychosocial Factors HRs were adjusted for age, sex, education, marital status, location, abdominal obesity, hypertension, smoking, diabetes, family history of CVD, and center random effects. No stress was used as the reference. CS-1 indicates low stress; CS-2, moderate stress; CS-3, high stress.

Separate analyses in men and women found that compared with no stress, the adjusted risk of death increased significantly with increasing levels of stress in men (low stress: HR. 1.14 [95% CI, 1.06-1.24]; high stress: 1.18 [95% CI, 1.02-1.36]) (Table 4). However, the association was only significant for women with medium level of stress compared with women with no stress (HR, 1.16 [95% CI, 1.05-1.28]). Men with high stress had higher risk of major CVD (HR, 1.38 [95% CI, 1.18-1.61]), major CHD (HR, 1.23 [95% CI, 1.02-1.48]) and stroke (HR, 1.55 [95% CI, 1.21-1.98]) compared with men with no stress. However, the associations were not significant in women, except for major CHD (Table 4). HR estimates for the association of global stress with outcomes by country income grouping were generally directionally similar to those of the total population but with wider CIs (eFigure 2 in the Supplement).

**Table 4.** Adjusted HRs of Composite Score of Psychosocial Factors and Outcomes in Men and Women

Outcome	Adjusted HR (95% CI)^a^	
	Men	Women
All-cause death		
Low stress	1.14 (1.06-1.24)	1.03 (0.94-1.12)
Medium stress	1.21 (1.10-1.33)	1.16 (1.05-1.28)
High stress	1.18 (1.02-1.36)	1.15 (1.00-1.32)
Major CVD		
Low stress	1.04 (0.95-1.13)	1.04 (0.94-1.14)
Medium stress	1.07 (0.96-1.20)	1.08 (0.96-1.21)
High stress	1.38 (1.18-1.61)	1.09 (0.92-1.30)
Major CHD		
Low stress	1.03 (0.93-1.15)	1.18 (1.06-1.33)
Medium stress	1.07 (0.94-1.22)	1.27 (1.10-1.45)
High stress	1.23 (1.02-1.48)	1.35 (1.11-1.64)
Stroke		
Low stress	1.11 (0.98-1.26)	0.97 (0.85-1.11)
Medium stress	1.12 (0.94-1.33)	0.99 (0.83-1.18)
High stress	1.55 (1.21-1.98)	1.07 (0.82-1.40)

Abbreviations: CHD, coronary heart disease; CVD, cardiovascular disease; HR, hazard ratio.

^a^ Adjusted for age, education, marital status, location, abdominal obesity, hypertension, smoking, diabetes, family history of CVD, and center random effects. No stress was used as the reference group.

## Discussion

In this large multiethnic prospective population-based cohort study from 21 countries at varying levels of economic development, we found a significant association between adverse psychosocial factors and increased risk of mortality, CVD, and stroke. Prior work from our group published in 2004 with the same measure for self-perceived stress^6^ found that individuals who experienced some periods and permanent stress at home had 1.45-fold higher risk for acute MI, and those with some periods or permanent stress at work experienced 2.17 higher risk. Estimates from pooled measures, as well as from single-item measures (general stress, life events over the past year, financial stress), from this prospective study found a directionally similar increase in risk, but the relative risks were smaller in size, which is consistent with prior studies.^3,4,19,20^ Because of the overall moderate association of psychosocial factors with mortality, CVD, and stroke, analysis by country income group was unreliable owing to reduced statistical power. Therefore, the primary emphasis should be on the overall analyses of our study.

Plausible explanations of a more modest association than that reported in our previous report^6^ include a longer follow-up period (median, 10.2 years), with a considerable interval between stress as an exposure and subsequent outcomes, which may have attenuated associations. In addition, individual response to stress may be influenced by prior life experiences or other response factors, such as psychological coping, resilience, and genetic factors,^21^ which were not included in the present study. Another potential explanation is that the risk of future incidence of disease or death depends on the cumulative exposures in everyday life to the conditions that elicit these responses, and it is the combination of exposure and how the individual responds and copes with it that is likely to be important.

Compared with other major risk factors, psychosocial variables are more difficult to define objectively because there is no consensus on how stress should be measured.^3,9^ It is likely that the perceived level of stress for the same exposure may differ from one person to another for a variety of individual and other factors. The subjective perception of psychological stress used in our study was measured using a single question that has a strong relationship with other psychological stressors, such as financial stress, stressful life events, and locus of control.^6^ Psychological stress was defined based on the degree to which respondents feel tense, irritable, or anxious or having sleeping difficulties. Although the stress measure may seem overly simple and measured only on one single occasion in midlife (age 35-70 years), it has been shown to be associated with cardiovascular events.^17,22^ Still, the concept of stress contained within the single question may be construed to cover a range of other factors, such as excessive work demand and job stress,^4,23^ adverse life events,^24^ financial problems,^25^ social isolation or reactions to stress (eg, depression),^26^ vigorous exhaustion, anxiety and psychological distress,^2,5,27^ and sleeping problems.^28,29^ Additionally, the perception of what constitutes stress likely varies across countries, ethnic groups, and cultural and individual socioeconomic factors and is subject to other contextual factors. Social norms on acceptance, recognition, and coping mechanism of stress differ among cultures, societies, and countries.^3,12^ Therefore, there is a need for the development assessment tools to ascertain stress across countries. To this end, combining several dimensions within the large concept of stress is likely a better approach than using single entities in this large and heterogeneous population. Analyses from individual countries showed directionally similar results, although with wide CIs, not allowing for firm conclusions.

Substantial evidence has accumulated with respect to the associations of stress and other psychosocial factors with CVD, mainly CHD and stroke,^9,30^ which is supported by our findings. How these factors should be assessed and quantified in a more reliable and reproducible manner across different settings remains to be determined. Adaptation to adverse circumstances, coping mechanisms, and mitigation are still underexplored. Therefore, future studies are needed to investigate stress exposure, stress responses, and potential mitigating or aggravating factors concurrently to determine more robust associations with future CVD, where data are still limited.^31^ The mechanisms by which psychological stress may influence outcomes are complex, since psychological stress is also often associated with deleterious behaviors, such as smoking, alcohol consumption, dietary choices, and lack of exercise, that may also affect the likelihood of CVD and premature death.^32,33^ However, we adjusted for these factors, so our results suggest an independent association between stress and outcomes. Although there is some evidence to support a link between psychological stress and CVD, the underlying mechanism by which stress increases CVD, which may involve the hypothalamus-pituitary-adrenal axis, sympathetic nervous system activation, and immunologic, neuroendocrine, and inflammatory processes, as well as the interaction of genetic and environmental factors,^1^ is still unclear. To address it, additional measures, such as biomarkers, are needed, which the PURE study is currently working to add to the collected data.

To our knowledge, this is the first prospective cohort study to describe a dose-response association between level of psychosocial factors (global stress, life events, and financial stress) and mortality and CVD risk in large cohort population-based sample across 21 countries at a wide range of economic levels, lifestyles, social circumstances, and cultures. This study also captured a relatively large number of incident events for analyses and thus has sufficient power to document associations between stress and future cardiovascular outcomes and mortality.

### Limitations

This study has some limitations. The simple questions used to measure psychosocial factors are partly subjective, but stress is a perception on the part of the individual. In this prospective design, there is less concern about bias or reverse causality, but the imprecision in the measure of exposure and the measurement at a single time point may dilute any real associations, so the real associations may be larger. The consistent direction of findings of the associations of life events (which may be more objective) with CVD and deaths provides reassurance that the findings we report are more likely to be real.

## Conclusions

This cohort study found that stress was independently associated with a higher risk of CVD and deaths. Our findings emphasize the need for the development and evaluation of prevention strategies to address whether modifying stress would reduce CVD. Further detailed and embedded mechanistic studies in large prospective studies conducted in different settings could boost understanding of how stress is associated with increased risk of CVD and deaths.

## Notes

Supplement.
eMethods
**eFigure 1.** Flowchart for the Definition of the Study Cohort **eFigure 2.** Adjusted Hazard Ratios of Composite Score of Psychosocial Factors and All-Cause Death, CVD, Coronary Heart Disease, and Stroke by Country Income **eTable 1.** Unadjusted and Adjusted Hazard Ratios of Composite Score of Psychosocial Factors and Outcomes for Complete Analysis and Multiple Imputed Data Analysis **eTable 2.** Baseline Socioeconomic Characteristics of PURE Participants by Global Stress **eTable 3.** Baseline Socioeconomic Characteristics of PURE Participants by Life Events **eTable 4.** Baseline Socioeconomic Characteristics of PURE Participants by Financial Stress **eTable 5.** Baseline Demographic Characteristics of PURE Participants by Global Stress **eTable 6.** Baseline Demographic Characteristics of PURE Participants by Life Events **eTable 7.** Baseline Demographic Characteristics of PURE Participants by Financial Stress
eReference
