# Omitted Author, Funding Information, and Study Group Members

**DOI:** 10.1001/jamanetworkopen.2022.10663

**Published:** 2022-04-13

In the Original Investigation titled “Psychosocial Risk Factors and Cardiovascular Disease and Death in a Population-Based Cohort From 21 Low-, Middle-, and High-Income Countries,”^1^ published December 15, 2021, the authors omitted a coauthor and funding information from the published article and misunderstood the process for listing nonauthor group members. Sadi Gulec, MD, should be listed as the fifth author, a list of nonauthor members of the Prospective Urban Rural Epidemiology (PURE) Study Group should have been published in a Supplement, and the following funding information should have been included in the Article Information section:

**Funding/Support:** Dr S. Yusuf is supported by the Marion W. Burke endowed chair of the Heart and Stroke Foundation of Ontario. The Prospective Urban Rural Epidemiology (PURE) study is an investigator-initiated study that is funded by the Population Health Research Institute, Hamilton Health Sciences Research Institute (HHSRI), the Canadian Institutes of Health Research, Heart and Stroke Foundation of Ontario, support from Canadian Institutes of Health Research’s Strategy for Patient Oriented Research (SPOR), through the Ontario SPOR Support Unit, as well as the Ontario Ministry of Health and Long-Term Care and through unrestricted grants from several pharmaceutical companies (with major contributions from AstraZeneca [Canada], Sanofi [France and Canada], Boehringer Ingelheim [Germany and Canada], Servier, and GlaxoSmithKline), and additional contributions from Novartis and King Pharma and from various national or local organizations in participating countries. These countries include Argentina: Fundacion ECLA (Estudios Clínicos Latino America); Bangladesh: Independent University, Bangladesh, and Mitra and Associates; Brazil: Hospital Alemão Oswaldo Cruz, São Paulo, Brazil; Canada: this study was supported by an unrestricted grant from Dairy Farmers of Canada and the National Dairy Council (US), Public Health Agency of Canada, and Champlain Cardiovascular Disease Prevention Network; Chile: Universidad de La Frontera (EXD05-0003); China: National Center for Cardiovascular Diseases and ThinkTank Research Center for Health Development; Colombia: Colciencias (grant No. 6566-04-18062 and 6517-777-58228); India: Indian Council of Medical Research; Malaysia: Ministry of Science, Technology and Innovation of Malaysia (grant No. 100-IRDC/BIOTEK 16/6/21 [13/2007] and 07-05-IFN-BPH 010), Ministry of Higher Education of Malaysia (grant No. 600-RMI/LRGS/5/3 [2/2011]), Universiti Teknologi MARA, Biostatistics & Data Repository Sector, National Institute of Health, Setia Alam (for the data linkage service), National Registration Department (JPN) (for their willingness to share their mortality records for research purposes), and Universiti Kebangsaan Malaysia (UKM-Hejim-Komuniti-15-2010); occupied Palestinian territory: the United Nations Relief and Works Agency for Palestine Refugees in the Near East, occupied Palestinian territory, and International Development Research Centre, Canada; Philippines: Philippine Council for Health Research and Development; Poland: Polish Ministry of Science and Higher Education (grant No. 290/W-PURE/2008/0) and Wroclaw Medical University; Saudi Arabia: Saudi Heart Association, Dr Mohammad Alfagih Hospital, the Deanship of Scientific Research at King Saud University (Research group No. RG -1436-013), Riyadh, and Saleh Hamza Serafi Chair for Research of Coronary Heart Disease, Umm AlQura University, Makkah, Saudi Arabia; South Africa: the North-West University, South Africa, and Netherlands Programme for Alternative Development, National Research Foundation, Medical Research Council of South Africa, the South Africa Sugar Association, and Faculty of Community and Health Sciences; Sweden: grants from the Swedish state under the Agreement Concerning Research and Education of Doctors; the Swedish Heart and Lung Foundation; the Swedish Research Council; the Swedish Council for Health, Working Life and Welfare, King Gustaf V and Queen Victoria Freemason’s Foundation, and AFA Insurance; Turkey: Metabolic Syndrome Society, AstraZeneca, and Sanofi; United Arab Emirates: Sheikh Hamdan Bin Rashid Al Maktoum Award For Medical Sciences and Dubai Health Authority, Dubai.

**Role of the Funder/Sponsor:** The funders had no role in the design and conduct of the study; collection, management, analysis, and interpretation of the data; preparation, review, or approval of the manuscript; and decision to submit the manuscript for publication.

The article has been corrected to include the omitted author’s name, affiliation, and contributions; a Supplement with the members of the Prospective Urban Rural Epidemiology (PURE) Study Group; and the missing funding information.^1^
