# Supplementary material for: Psychosocial Risk Factors and Cardiovascular Disease and Death in a Population-Based Cohort From 21 Low-, Middle-, and High-Income Countries
Source: JAMA Netw Open. 2021 Dec 15;4(12):e2138920. doi: 10.1001/jamanetworkopen.2021.38920 (PMC8674745; doi:10.1001/jamanetworkopen.2021.38920)
Supplement: Supplement. — eMethods eFigure 1. Flowchart for the Definition of the Study Cohort eFigure 2. Adjusted Hazard Ratios of Composite Score of Psychosocial Factors and All-Cause Death, CVD, Coronary Heart Disease, and Stroke by Country Income eTable 1. Unadjusted and Adjusted Hazard Ratios of Composite Score of Psychosocial Factors and Outcomes for Complete Analysis and Multiple Imputed Data Analysis eTable 2. Baseline Socioeconomic Characteristics of PURE Participants by Global Stress eTable 3. Baseline Socioeconomic Characteristics of PURE Participants by Life Events eTable 4. Baseline Socioeconomic Characteristics of PURE Participants by Financial Stress eTable 5. Baseline Demographic Characteristics of PURE Participants by Global Stress eTable 6. Baseline Demographic Characteristics of PURE Participants by Life Events eTable 7. Baseline Demographic Characteristics of PURE Participants by Financial Stress eReference [file jamanetwopen-e2138920-s001.pdf]

Supplemental Online Content

Santosa A, Rosengren A, Ramasundarahettige C, et al. Psychosocial risk factors and cardiovascular disease and death in a population-based cohort from 21 low-, middle-, and high-income countries. *JAMA Netw Open*. 2021;4(12):e2138920. doi:10.1001/jamanetworkopen.2021.38920

eMethods
eFigure 1. Flowchart for the Definition of the Study Cohort
eFigure 2. Adjusted Hazard Ratios of Composite Score of Psychosocial Factors and All-Cause Death, CVD, Coronary Heart Disease, and Stroke by Country Income
eTable 1. Unadjusted and Adjusted Hazard Ratios of Composite Score of Psychosocial Factors and Outcomes for Complete Analysis and Multiple Imputed Data Analysis
eTable 2. Baseline Socioeconomic Characteristics of PURE Participants by Global Stress
eTable 3. Baseline Socioeconomic Characteristics of PURE Participants by Life Events
eTable 4. Baseline Socioeconomic Characteristics of PURE Participants by Financial Stress
eTable 5. Baseline Demographic Characteristics of PURE Participants by Global Stress
eTable 6. Baseline Demographic Characteristics of PURE Participants by Life Events
eTable 7. Baseline Demographic Characteristics of PURE Participants by Financial Stress
eReference

This supplemental material has been provided by the authors to give readers additional information about their work.

© 2021 Santosa A et al. *JAMA Network Open.*

eMethods

PURE Study Design

The choice and number of countries selected in the Prospective Urban Rural Epidemiological Study (PURE Study) involves a large number of communities in countries at different economic levels, with substantial heterogeneity in social and economic circumstances and policies, with the feasibility of centers to successfully achieve long-term follow-up being a key factor. Thus, PURE included sites in which investigators are committed to collecting good-quality data for a low-budget study over the planned 10-year follow-up period without a strict proportionate sampling. The PURE study enrolled 202,123 individuals from 21 low, middle and high-income countries among whom there was baseline and follow-up data for 160,697 individuals aged 35 to 70 years from 21 countries. After exclusions of 29,784 individuals on account of missing data for key variables (eFigure S1) the study population for the present investigation includes 118,706 individuals, sampled from 612 communities from 21 countries from 5 continents representing a broad range of economic and social circumstances. PURE includes countries in four income strata based on World Bank classification in 2006: five low-income countries (Bangladesh, India, Pakistan, Tanzania, and Zimbabwe), twelve middle-income countries (Argentina, Brazil, Chile, China, Colombia, Iran, Malaysia, Palestine, Poland, South Africa, Turkey, Philippines), and four high-income countries (Canada, Saudi Arabia, Sweden, and United Arab Emirates) (see eFigure S1). The study is coordinated by the Population Health Research Institute, Hamilton Health Sciences and McMaster University, Canada.

Prospective Follow-up for Cardiovascular Events and Mortality

History of disease was collected at baseline from every participant with standardized questionnaires. Follow-up was initiated in all sites by March 2004 and completed by March, 2021. Up to three attempts were made to interview all households to document events. Information on specific events (death, myocardial infarction, stroke, heart failure, cancer, hospitalizations, new diabetes, injury, tuberculosis, human immunodeficiency viral infections, malaria, pneumonia, asthma, chronic obstructive pulmonary disease) were obtained from participants or their family members. This information was adjudicated centrally in each country by trained physicians using standardized definitions. Because the PURE study involves urban and rural areas from middle- and low-income countries, supporting documents to confirm cause of death and/or event varied in degrees of completion and availability. In most of middle- and low-income countries there was no central system of death or event registration. Therefore, information was obtained about prior medical illness and medically certified cause of death where available, and second, best available information was captured from reliable sources in those instances where medical information was not available in order to be able to arrive at a probable diagnosis or cause of death. Event documentation was based on information from household interviews and medical records, death certificates and other sources. Verbal autopsies were also used to ascertain cause of death in addition to medical records which were reviewed by a health professional. This approach has been used in several studies conducted in middle- and low-income countries[1].

To ensure a standard approach and accuracy for classification of events across all countries and over time, the first 100 CVD events (deaths, MI, strokes, heart failure or cancers) for China and India, and 50 cases for other countries were adjudicated both locally and also by the adjudication chair, and if necessary further training was provided. Thereafter, every year, 50 cases for China and India and 25 cases for each of the remaining countries were adjudicated as above.

Event Definitions

FATAL EVENTS

Cardiovascular Death – Definitions

01.00 DEATH DUE TO CARDIOVASCULAR EVENTS

01.10 Sudden unexpected Cardiovascular Death (SCVD)

Without evidence of other cause of death, death that occurred suddenly and unexpectedly (examples: witnessed collapse, persons resuscitated from cardiac arrest who later died) or persons seen alive less than 12 hours prior to discovery of death (example persons found dead in his/her bed).

- SCVD is either definite, probable or possible according to the following characteristics:

PURE Adjudication Code	Event Type	Acceptable ICD-10 codes
**01.11: Definite**	One of the following in persons with:<br>• known cardiovascular disease, or	

© 2021 Santosa A et al. *JAMA Network Open.*

	• diabetes with an additional risk factor such as hypertension, smoking, dyslipidemia, micro albuminuria, serum creatinine 50% above upper limit of normal, or<br>• 3 of the above risk factors, or<br>• 2 of the above risk factors in men aged 60 and more and women aged 65 and more	No ICD-10 Code
**01.12: <u>Probable</u>**	One of the following in persons with:<br>• diabetes, or<br>• 2 of the above risk factors in men aged less than 60 and in women less than 65, or<br>• one of the above risk factors in men aged 60 and more and in women aged 65 and more, or<br>• typical of chest pain or sudden severe dyspnea of less than 20-minute duration preceding the event	
**01.13: <u>Possible</u>**	In persons without risk factor	
*For **SCVD**, the patient was well or had a stable CVD (example stable angina) when last seen alive. The event of a sudden death occurring during the hospitalization of MI is considered a fatal MI and not sudden death.*		

01.30 Fatal Myocardial Infarction
Symptoms of Myocardial Infarction:
Typical symptoms or suggestive symptoms of MI according to physician are characterized by severe anterior chest pain as tightness, crushing, burning, lasting at least 20 minutes, occurring at rest, or on exertion, that may radiate to the arms or neck or jaw and may be associated with dyspnea, diaphoresis and nausea. However, death associated with nausea and vomiting with or without chest pain not due to another cause may be considered as possible MI if ECG and cardiac markers are not done. These symptoms may have occurred the last month before death.
Fatal myocardial infarction is either definite, probable or possible according to the following characteristics:

PURE Adjudication Code	Event Type	Acceptable ICD-10 codes
**01.31: <u>Definite</u>**	1. Autopsy demonstrating fresh myocardial infarction and/or recent coronary occlusion, **or**<br>2. ECG showing new and definite sign of MI (Minnesota code 1-1-1) **or**<br>3. Symptoms typical or atypical or inadequately described but attributed to cardiac origin lasting at least 20 minutes and by troponin or cardiac enzymes (CKMB, CK, SGOT, SLDH) above center laboratory ULN<br>4. ECG with new ischemic changes (new ST elevation/depression or T wave inversion ≥ 2 mm) and by troponin or cardiac enzymes (CKMB, CK, SGOT, SLDH) above center laboratory ULN	I21- I22
**01.32: <u>Probable</u>**	1. ECG with sign of probable MI (Minnesota code 1-2-1), **or**<br>2. Typical symptoms lasting at least 20 minutes considered of cardiac origin, with only new ST-T changes (new ST elevation/depression or T wave inversion ≥ 1 but < 2mm) without documented increased cardiac markers or enzyme as in PURE definition 1.31 (above), **or**<br>3. Increased cardiac enzymes as in PURE definition 1.31 (above) showing a typical pattern of MI as above without symptoms or significant ECG changes	
**01.33: <u>Possible</u>**	1. ECG with sign of possible MI (Minnesota code 1-3-1) **or**<br>2. Typical symptoms or symptoms suggestive of MI according to the physician lasting at least 20 minutes without documented ECG or cardiac marker.	

The **Minnesota codes** for MI is taken from Rose and Blackburn and published in their book "Evaluation Methods of Cardiovascular Disease WHO 1969".

- **Definite MI** is Q/R ratio ≥1/3 and Q duration ≥ 0.03 second in one of the following leads: I, II, V2, 3, 4, 5, 6. (code 1-1-1)
- **Probable MI** is Q/R ratio ≥1/3 and Q duration between 0.02 and 0.03 second in one of the following leads: I, II, V2, 3, 4, 5, 6. (code 1-2-1)

© 2021 Santosa A et al. *JAMA Network Open.*

- **Possible MI** is Q/R ratio between 1/5 and 1/3 and Q duration between 0.02 and 0.03 second in one of the following leads: I, II, V2, 3, 4, 5, 6. (code 1-3-1)

01.40 Fatal Stroke
Fatal stroke is either definite or possible according to the following characteristics:

PURE Adjudication Code	Event Type	Acceptable ICD-10 codes
**01.41: <u>Definite</u>**	Stroke death is defined as death within 30 days from an acute focal neurological deficit ***diagnosed by a physician*** and thought to be of vascular origin (without other cause such as brain tumor) with signs and symptoms lasting >= 24 hrs.<br><br>Stroke death is also considered if death occurred within 24 hrs. of onset of persisting signs and symptoms, or if there is evidence of a recent stroke on autopsy.<br>N.B.<br>• **In a subject with a stroke <= 30 days**: If death occurred with a pneumonia due to possible aspiration, death will be considered to be due to stroke.<br>• **In a subject with a stroke > 30 days**: If death occurred with a pneumonia due to possible aspiration, the adjudicator will make a decision according to his/her clinical judgment if death is related to stroke or not.<br>• Subarachnoid hemorrhage death manifested by sudden onset headache with/without focal signs and imaging (CT or MRI) evidence of bleeding primarily in the subarachnoid space is considered a fatal stroke in absence of trauma or brain tumor or malformation<br><br>• Subdural hematoma death is <u>not</u> considered as a stroke death and may be related to previous trauma or other cause.	I60- I64, I69
**01.43: <u>Possible</u>**	Death in a participant with a history of sudden onset of focal neurological deficit of one or more limbs, loss of vision or slurred speech lasting about 24 hours.	

01.50 Fatal Congestive Heart Failure
Fatal congestive heart failure is either definite or possible according to the following characteristics:

PURE Adjudication Code	Event Type	Acceptable ICD-10 codes
**01.51: <u>Definite</u>**	The diagnosis of congestive heart failure may be an autopsy finding in absence of other cause or requires signs (rales, increased jugular venous pressure or ankle edema) or symptoms (nocturnal paroxysmal dyspnea, dyspnea at rest or ankle edema) of congestive heart failure **<u>and</u>** one or both of the following:<br>• radiological signs of pulmonary congestion,<br>• treatment of heart failure with diuretics<br>*If sudden death occurred in a patient with chronic severe heart failure, it should be adjudicated as fatal congestive heart failure.*	I50
**01.52: <u>Probable</u>**	Progressive shortness of breath on lying down or at night, improving on sitting up AND any of the following signs or symptoms: swelling of feet, distension of abdomen, progressive cough in a person with known hypertension or a history of previous MI/angina or other heart disease	
**01.53: <u>Possible</u>**	Progressive shortness of breath on lying down or at night, improving on sitting up AND any of the following signs or symptoms: swelling of feet, distension of abdomen, progressive cough	

© 2021 Santosa A et al. *JAMA Network Open.*

01.60 Death Due to Other Cardiovascular Deaths *(other causes [1.10 to 1.50 above] having been excluded)*

PURE Adjudication Code	Event Type	Acceptable ICD-10 codes
**01.61**	Arterial rupture of aneurysm	I71- I72
**01.62**	Pulmonary embolism<br>*NOTE: Death associated with pulmonary embolism occurring* ***<u>within 2 weeks</u>*** *after a fracture such as hip, femur should attribute to death due to injury. Refer to Injury, Section 6.0*	I26
**01.63**	Arrhythmic death (A-V block, sustained ventricular tachycardia in absence of other causes)	I44- I45, I47- I49
**01.64**	Death after invasive cardiovascular intervention: a perioperative death extending to 30 days after coronary or arterial surgical revascularization and to 7 days after a coronary or arterial percutaneous dilatation (angioplasty) with or without a stent or an invasive diagnostic procedure.	I97
**01.65**	Congenital heart disease	Q20-Q28
**01.66**	Heart valve disease (including rheumatic heart disease)	I01, I05- I09, I34- I37
**01.67**	Endocarditis	I33, I38
**01.68**	Myocarditis	I40
**01.69**	Tamponade (pericarditis)	I30, I31, I32
**01.70**	Other cardiovascular events *(Excluding* ***1.61 to 1.69*** *above)*<br>***Valid*** *ICD-10 codes would include the following:*<br>*I11, I12, I13, I23, I24, I25, I27, I28, I42, I51, I52, I65-I68, I73, I74, I96, I98, I99 (Refer to ICD-10 Listing for associated definitions for each code)*	Any valid 'I' (Cardiovascular) ICD-10 code that can be classified as underlying cause of death, not specified above

NON-FATAL EVENTS
Cardiovascular Events – Definitions
<u>10.00 NON-FATAL CARDIOVASCULAR EVENTS</u>
10.10 Non-Periprocedural Myocardial Infarction (MI)

MI is considered either definite, probable or possible according to the following characteristics:

PURE Adjudication Code	Event Type	Acceptable ICD-10 codes
**10.11: <u>Definite</u>**	1. ECG showing new and definite sign of MI (Minnesota code 1-1-1) **or**<br>2. Symptoms typical or atypical or inadequately described but attributed to cardiac origin lasting at least 20 minutes and by troponin or cardiac enzymes (CKMB, CK, SGOT, SLDH) above center laboratory ULN	

© 2021 Santosa A et al. *JAMA Network Open.*

	3. ECG with new ischemic changes (new ST elevation/depression or T wave inversion ≥ 2 mm) and by troponin or cardiac enzymes (CKMB, CK, SGOT, SLDH) above center laboratory ULN<br><br>Please note that increased markers may occur in trauma (CK, AST, myoglobin and CK MB to a lesser degree); renal insufficiency, heart failure, pulmonary embolism…. (troponin), cardioversion (all)	I21-I22
**10.12: <u>Probable</u>**	1. ECG with new and probable sign of MI (Minnesota code 1-2-1), **or**<br>2. Typical symptoms lasting at least 20 minutes considered of cardiac origin, with only new ST-T changes (new ST elevation/depression or T wave inversion ≥ 1 but < 2mm) without documented increased cardiac markers as in PURE definition 10.11 (above), **or**<br>3. Increased cardiac enzymes showing a typical pattern of MI as above without symptoms or significant ECG changes.	
**10.13: <u>Possible</u>**	1. ECG with new and possible sign of MI (Minnesota code 1-3-1), **or**<br>2. Typical symptoms lasting 20 minutes and more considered to be of cardiac origin without documented ECG or cardiac marker.	

10.20 Periprocedural Myocardial Infarction

PURE Adjudication Code	Event Type	Acceptable ICD-10 codes
**10.21: <u>Definite</u>**	1. ECG showing new and definite sign of MI (Minnesota code 1-1-1), or<br>2. Increased cardiac markers within 48 hours of procedure:<br>• percutaneous coronary intervention: CKMB should be ≥ 5 X ULN or troponin ≥ 5 X above lower level of necrosis **OR** > 20% increase in cardiac markers if elevated at the beginning of the procedure in a patient with symptoms suggestive of myocardial ischemia<br>• Coronary surgery: Increased cardiac markers CKMB should be ≥ 10X ULN or troponin ≥ 10X above lower limit of necrosis.	I21-I22

The **Minnesota codes** for MI is taken from Rose and Blackburn and published in their book "Evaluation Methods of Cardiovascular Disease WHO 1969".

- **Definite MI** is Q/R ratio ≥1/3 and Q duration ≥ 0.03 second in one of the following leads: I, II, V2, 3, 4, 5, 6. (code 1-1-1)
- **Probable MI** is Q/R ratio ≥1/3 and Q duration between 0.02 and 0.03 second in one of the following leads: I, II, V2, 3, 4, 5, 6. (code 1-2-1)
- **Possible MI** is Q/R ratio between 1/5 and 1/3 and Q duration between 0.02 and 0.03 second in one of the following leads: I, II, V2, 3, 4, 5, 6. (code 1-3-1)

10.30 Stroke/Transient Ischemic Attack (TIA)

PURE Adjudication Code	Event Type	Acceptable ICD-10 codes

© 2021 Santosa A et al. *JAMA Network Open.*

**10.31: <u>Definite</u>**	Stroke is defined as an acute focal neurological deficit ***diagnosed by a physician*** and thought to be of vascular origin (without other case such as brain tumor) with signs and symptoms lasting ≥ 24 hrs.<br><br>N.B.<br>• Subarachnoid hemorrhage manifested by sudden onset headache with/without focal signs and imaging (CT or MRI or lumbar puncture) showing evidence of bleeding primarily in the subarachnoid space is considered a stroke in absence of trauma or brain tumor or malformation<br>• Subdural hematoma is <u>not</u> considered as a stroke and may be related to previous trauma or other cause.	I60-I64, I69
**10.33: <u>Possible</u>**	Stroke is possible if there is a history of sudden onset of focal neurological deficit of one or more limbs, loss of vision or slurred speech lasting about 24 hours or more	
**10.34: <u>TIA</u>**	The diagnosis of TIA requires the presence of acute focal neurological deficit thought to be of vascular origin with signs and symptoms lasting less than 24 hours	G45

10.40 Congestive Heart Failure

PURE Adjudication Code	Event Type	Acceptable ICD-10 codes
**10.41: <u>Definite</u>**	The diagnosis of congestive heart failure requires signs (rales, increased jugular venous pressure or ankle edema) or symptoms (nocturnal paroxysmal dyspnea, dyspnea at rest or ankle edema) of congestive heart failure **<u>and</u>** one or both of the following:<br>• radiological signs of pulmonary congestion,<br>• Treatment of heart failure with diuretics.	I50
**10.42: <u>Probable</u>**	Progressive shortness of breath on lying down or at night, improving on sitting up AND any of the following signs or symptoms: swelling of feet, distension of abdomen, progressive cough in a person with known hypertension or a history of previous MI/angina or other heart disease	
**10.43: <u>Possible</u>**	Congestive heart failure is considered possible when there is progressive shortness of breath on lying down or at night, improving on sitting up AND any of the following signs or symptoms: swelling of feet, distension of abdomen, progressive cough	

10.50 Effort Angina with documented Ischemia

PURE Adjudication Code	Event Type	Acceptable ICD-10 codes

© 2021 Santosa A et al. *JAMA Network Open.*

**10.51: Definite**	Stress test with ECG with new ST depression >1 mm or positive imaging (ECHO, Scan) compatible with ischemia	I20
**10.52: Probable**	Typical effort angina (i.e. Squeezing, pressure or burning type pain touching the sternum occurring on exertion and relieved by rest or nitroglycerin)	

10.60 Unstable Angina

PURE Adjudication Code	Event Type	Acceptable ICD-10 codes
**10.61: <u>Definite</u>**	1. Hospitalization for typical symptoms with new ECG changes (T wave inversion > 2mm) **<u>OR</u>**<br>2. Coronary revascularization within one week of admission and treated as unstable angina.	I20
**10.62: <u>Probable</u>**	1. Hospitalization for typical symptoms lasting at least 10 but less than 20 minutes without ECG or cardiac enzyme elevation	
**10.63: <u>Possible</u>**	Not hospitalized for typical symptoms of angina occurring at rest and treated as unstable angina: rest, anti-platelets, nitrates, beta blockers and/or calcium channel blockers.	

10.80 Other Non-Fatal Cardiovascular Events

PURE Adjudication Code	Event Type	Acceptable ICD-10 codes
**10.81**	Rheumatic Heart Disease	I01, I05-I09
**10.82**	Other valvular heart diseases (excluding Rheumatic Heart Disease)	I34-I37
**10.83**	Pericarditis	I30, I31, I32
**10.84**	Endocarditis	I33, I38
**10.85**	Myocarditis	I40
**10.86**	Congenital heart disease	Q20-Q28
**10.87**	Atrial fibrillation/flutter<br>Atrial Fibrillation and Flutter are due to an abnormal cardiac rhythm at the atrial level, and the diagnosis is made on the electrocardiographic (ECG) tracing or monitor.	I48
**10.88**	Peripheral artery disease (lower limb iliac to popliteal and carotid)	I73
**10.90**	Pulmonary embolism	I26
**10.91**	Other cardiac or arterial diseases:<br>*Specific details should be provided on the corresponding Adjudication Form*	Any ‘I’ (Cardiovascular) ICD-10 code not classified above

© 2021 Santosa A et al. *JAMA Network Open.*

eFigure 1. Flowchart for the Definition of the Study Cohort

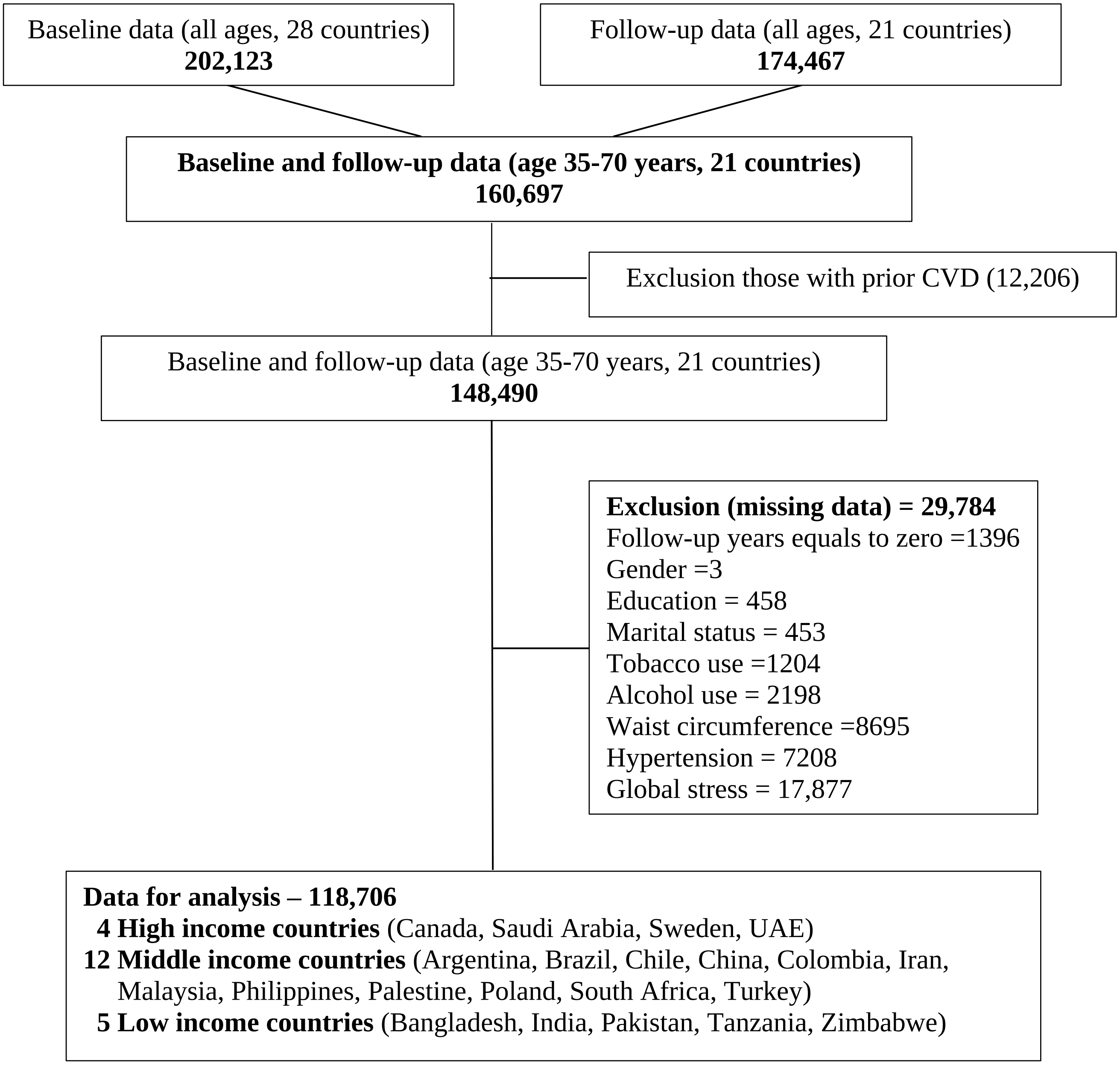

© 2021 Santosa A et al. *JAMA Network Open.*

eFigure 2. Adjusted* Hazard Ratios of Composite Score of Psychosocial Factors and All-Cause Death, CVD, Coronary Heart Disease, and Stroke by Country Income
eFigure 2a.

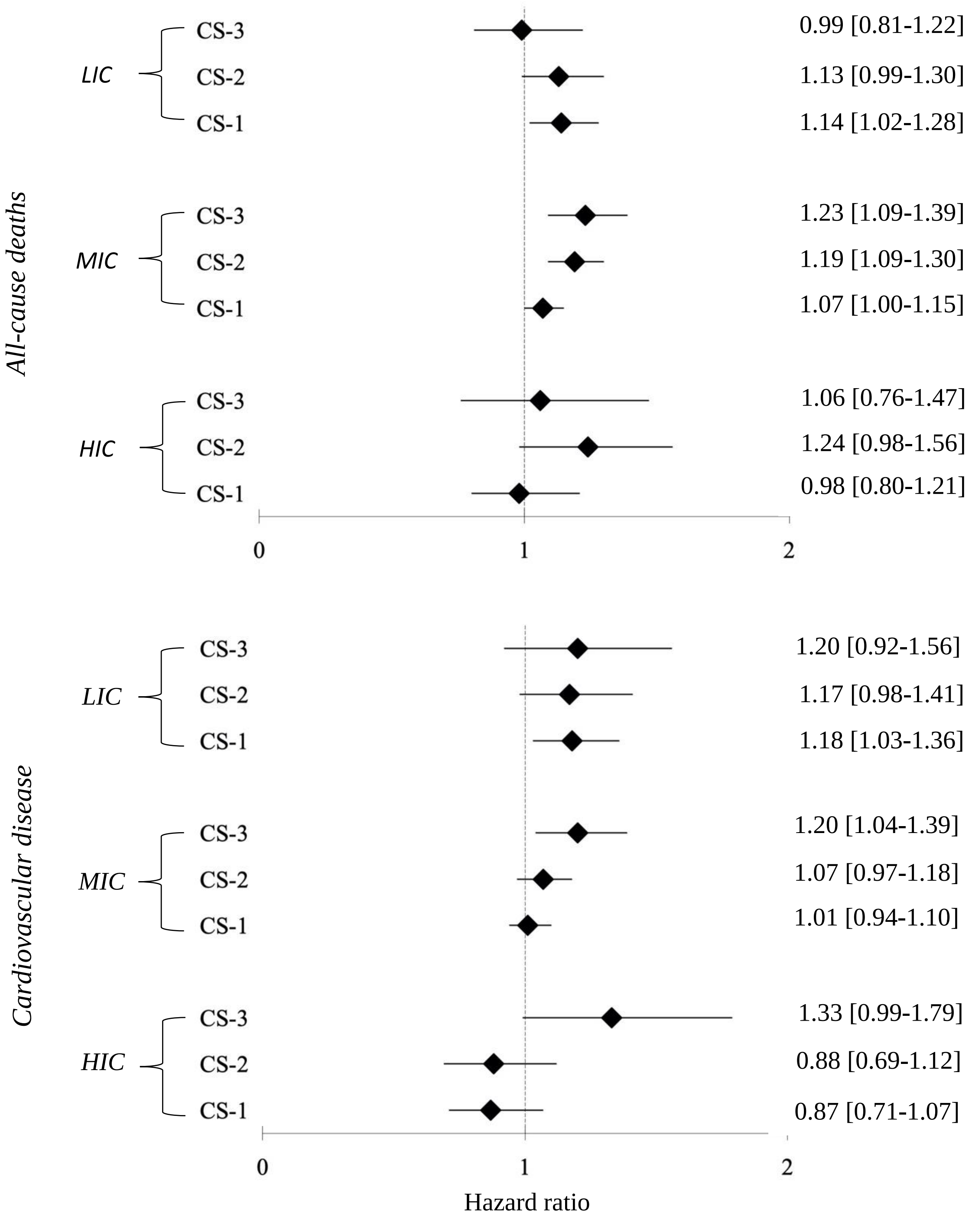

CS-1 low stress; CS-2 moderate stress, CS-3 high stress

*Adjusted for age, gender, education, marital status, location, abdominal obesity, hypertension, smoking, diabetes, family history of CVD and center random effects.
Foot notes: CS-0 no stress as reference group.
HIC: High Income Countries, MIC: Middle Income Countries; LIC: Low Income Countries

© 2021 Santosa A et al. *JAMA Network Open.*

eFigure 2b.

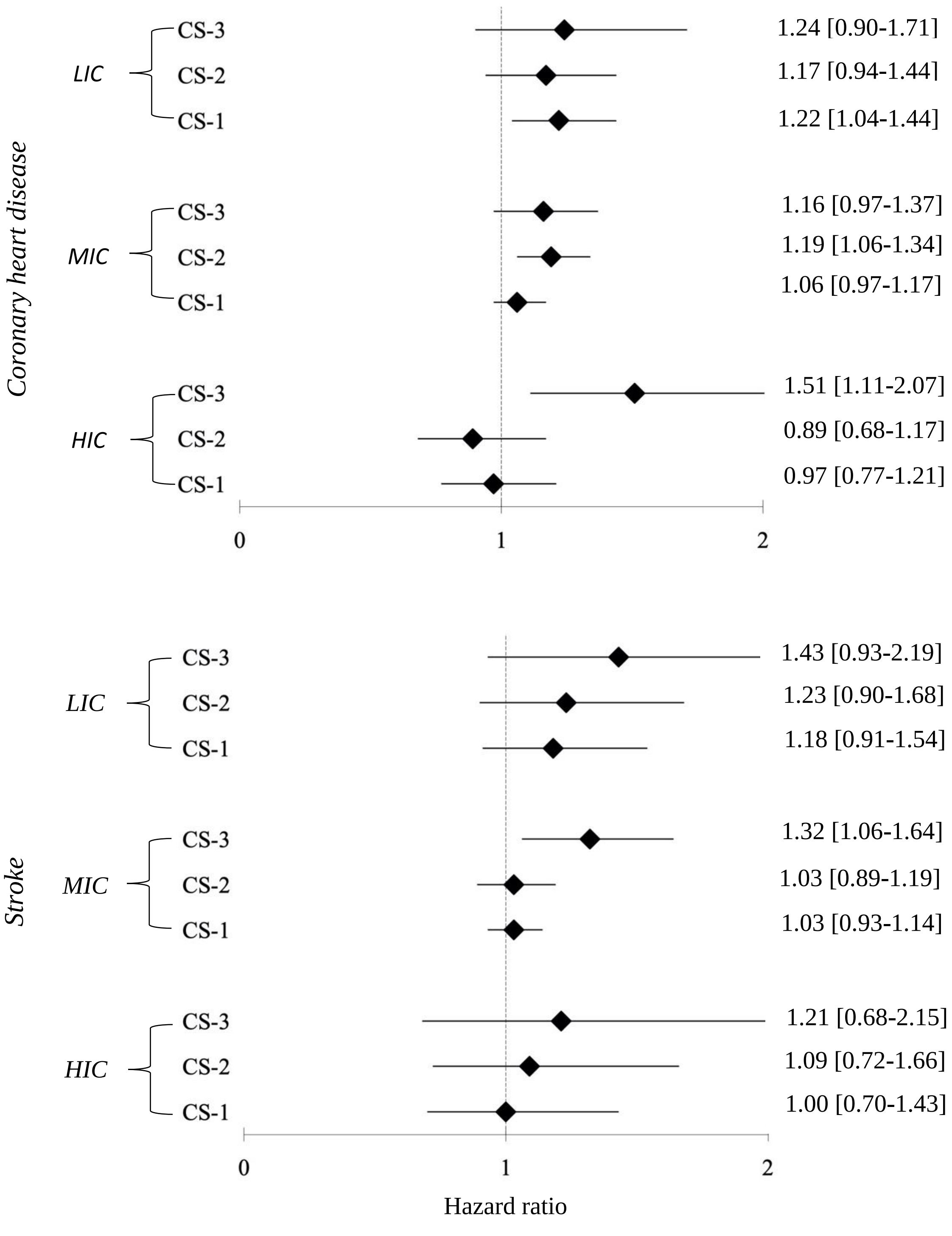

CS-1 low stress; CS-2 moderate stress, CS-3 high stress

*Adjusted for age, gender, education, marital status, location, abdominal obesity, hypertension, smoking, diabetes, family history of CVD and center random effects.

Foot notes: CS-0 no stress as reference group.

HIC: High Income Countries, MIC: Middle Income Countries; LIC: Low Income Countries

© 2021 Santosa A et al. *JAMA Network Open.*

eTable 1. Unadjusted and Adjusted Hazard Ratios of Composite Score of Psychosocial Factors and Outcomes for Complete Analysis and Multiple Imputed Data Analysis

Outcomes	Composite score of stress	Complete analysis		Analysis of imputed data	
		Unadjusted HR [95% CI]	Adjusted* HR [95% CI]	Unadjusted HR [95% CI]	Adjusted* HR [95% CI]
All-deaths	Low stress	1.06 [1.00-1.12]	1.09 [1.03-1.16]	1.07 [1.02-1.12]	1.06 [1.01-1.11]
	Medium stress	1.06 [0.99-1.14]	1.19 [1.11-1.27]	1.07 [1.00-1.13]	1.16 [1.09-1.23]
	High stress	0.96 [0.87-1.06]	1.17 [1.06-1.29]	1.01 [0.93-1.11]	1.17 [1.07-1.28]
CVD	Low stress	0.99 [0.94-1.04]	1.06 [1.01-1.12]	0.98 [0.93-1.04]	1.03 [0.97-1.09]
	Medium stress	0.98 [0.92-1.04]	1.13 [1.06-1.20]	0.96 [0.89-1.04]	1.09 [1.01-1.17]
	High stress	1.01 [0.92-1.10]	1.24 [1.14-1.36]	1.04 [0.93-1.16]	1.24 [1.11-1.39]
Major CHD	Low stress	1.04 [0.96-1.12]	1.09 [1.01-1.18]	1.05 [0.98-1.12]	1.09 [1.02-1.16]
	Medium stress	1.03 [0.94-1.13]	1.15 [1.04-1.26]	1.06 [0.97-1.16]	1.17 [1.07-1.28]
	High stress	1.06 [0.92-1.21]	1.24 [1.08-1.42]	1.08 [0.96-1.23]	1.27 [1.11-1.44]
Stroke	Low stress	1.10 [0.91-1.31]	1.05 [0.96-1.15]	0.96 [0.89-1.04]	1.03 [0.95-1.12]
	Medium stress	1.10 [0.87-1.39]	1.07 [0.94-1.21]	0.92 [0.82-1.03]	1.07 [0.95-1.20]
	High stress	1.10 [0.79-1.55]	1.30 [1.09-1.56]	1.07 [0.90-1.27]	1.32 [1.11-1.57]

*Adjusted for age, gender, education, marital status, location, abdominal obesity, hypertension, smoking, diabetes, family history of CVD and center random effects.
Foot notes: No stress as reference group

© 2021 Santosa A et al. *JAMA Network Open.*

eTable 2. Baseline Socioeconomic Characteristics of PURE Participants by Global Stress

Variables	Never	≥1 period	Several periods	Permanent
**Country income**				
High income	2370 (15.2)	7431 (47.7)	4671 (30.0)	1116 (7.2)
Middle income	35737 (41.1)	36120 (41.6)	9466 (10.9)	5614 (6.5)
Low income	6290 (38.9)	7962 (49.2)	1668 (10.3)	261 (1.6)
**Education**				
None/primary school	17475 (36.3)	21402 (44.5)	6283 (13.1)	2957 (6.1)
Secondary/High school	19737 (43.2)	19159 (42.0)	4518 (9.9)	2222 (4.9)
College/ University	7185 (28.8)	10952 (43.9)	5004 (20.0)	1812 (7.3)
**Marital status**				
Never married	1288 (23.3)	2441 (44.1)	1261 (22.8)	544 (9.8)
Currently married/living with partner	39856 (39.4)	44008 (43.2)	12293 (12.1)	5417 (5.3)
Widowed/Divorced	3253 (28.0)	5064 (43.7)	2251 (19.4)	1030 (8.9)
**Area**				
Rural area	21884 (39.1)	24871 (44.4)	6611 (11.8)	2615 (4.7)
Urban area	22513 (35.9)	26642 (42.5)	9194 (14.6)	4376 (7.0)

© 2021 Santosa A et al. *JAMA Network Open.*

eTable 3. Baseline Socioeconomic Characteristics of PURE Participants by Life Events

Variables	None	One life event	Two/more life events
**Country income**			
High income	8055 (51.7)	4570 (29.3)	2963 (19.0)
Middle income	61730 (71.0)	16481 (19.0)	8726 (10.0)
Low income	11135 (68.8)	3288 (20.3)	1758 (10.9)
**Education**			
None/primary school	31592 (65.7)	10512 (21.8)	6013 (12.5)
Secondary/High school	33681 (73.8)	8056 (17.7)	3899 (8.5)
College/ University	15647 (62.7)	5771 (23.1)	3535 (14.2)
**Marital status**			
Never married	2926 (52.9)	1514 (27.4)	1094 (19.8)
Currently married/living with partner	71724 (70.6)	19956 (19.7)	9894 (9.7)
Widowed/Divorced	6270 (54.1)	2869 (24.7)	2459 (21.2)
**Area**			
Rural area	39451 (70.5)	11281 (20.1)	5249 (9.4)
Urban area	41469 (66.1)	13058 (20.8)	8198 (13.1)

© 2021 Santosa A et al. *JAMA Network Open.*

eTable 4. Baseline Socioeconomic Characteristics of PURE Participants by Financial Stress

Variables	None/little	Moderate	High/severe	a
**Country income**				
High income	9852 (63.2)	3975 (25.5)	895 (5.7)	866 (5.6)
Middle income	51215 (58.9)	23638 (27.2)	7782 (9.0)	4302 (4.9)
Low income	7520 (46.5)	5870 (36.3)	1907 (11.8)	884 (5.4)
**Education**				
None/primary school	24778 (51.5)	15183 (31.6)	5696 (11.8)	2460 (5.1)
Secondary/High school	28060 (61.5)	11969 (26.2)	3306 (7.3)	2301 (5.0)
College/ University	15749 (63.1)	6331 (25.4)	1582 (6.3)	1291 (5.2)
**Marital status**				
Never married	2381 (43.0)	1867 (33.7)	963 (17.4)	323 (5.8)
Currently married/living with partner	61103 (60.2)	27492 (27.1)	7739 (7.6)	5240 (5.2)
Widowed/Divorced	5103 (44.0)	4124 (35.6)	1882 (16.2)	489 (4.2)
**Area**				
Rural area	31653 (56.5)	16425 (29.3)	4981 (8.9)	2922 (5.2)
Urban area	36934 (58.9)	17058 (27.2)	5603 (8.9)	3130 (5.0)

[a] missing data

© 2021 Santosa A et al. *JAMA Network Open.*

eTable 5. Baseline Demographic Characteristics of PURE Participants by Global Stress

**Variables**	**Never**	**≥1 period**	**Several periods**	**Permanen t**
Age (mean, SD)	51.7 ± 9.8	49.9 ± 9.5	49.3 ± 9.0	48.9 ± 8.8
Male	19086 (43.0)	21201 (41.2)	6027 (38.1)	2550 (36.5)
Family history of CVD	17047 (38.4)	24361 (47.3)	9584 (60.6)	4284 (61.3)
Tobacco use				
Current smoker	9269 (20.9)	10456 (20.3)	3383 (21.4)	1834 (26.2)
Former smoker	3434 (7.7)	5903 (11.5)	2933 (18.6)	1077 (15.4)
Never smoke	31694 (71.4)	35154 (68.2)	9489 (60.0)	4080 (58.4)
Abdominal obesity	9024 (20.3)	13082 (25.4)	4932 (31.2)	2213 (31.6)
Alcohol use	11227 (25.3)	16587 (32.2)	7829 (49.5)	3182 (45.5)
Hypertension	19010 (42.8)	20510 (39.8)	6012 (38.0)	2745 (39.3)
Diabetes	1902 (4.3)	2456 (4.7)	757 (4.8)	323 (4.6)

© 2021 Santosa A et al. *JAMA Network Open.*

eTable 6. Baseline Demographic Characteristics of PURE Participants by Life Events

**Variables**	**None**	**One life event**	**Two/more life events**
Age (mean, SD)	50.5 ± 9.7	50.4 ± 9.4	50.4 ± 9.4
Male	33650 (41.6)	9938 (40.8)	5276 (39.2)
Family history of CVD	33369 (41.2)	13629 (56.0)	8278 (61.6)
Tobacco use			
Current smoker	16589 (20.5)	5149 (21.2)	3204 (23.8)
Former smoker	7137 (8.8)	3699 (15.2)	2511 (18.7)
Never smoke	57194 (70.7)	15491 (63.6)	7732 (57.5)
Abdominal obesity	17651 (21.8)	7072 (29.1)	4528 (33.7)
Alcohol use	22791 (28.2)	9536 (39.2)	6498 (48.3)
Hypertension	33063 (40.9)	9755 (40.1)	5459 (40.6)
Diabetes	3511 (4.3)	1232 (5.1)	695 (5.2)

© 2021 Santosa A et al. *JAMA Network Open.*

eTable 7. Baseline Demographic Characteristics of PURE Participants by Financial Stress

Variables	None/little	Moderate	High/severe	a
Age (mean, SD)	51.0 ± 9.7	49.4± 9.4	49.3± 9.1	51.8 ± 9.6
Male	29204 (42.6)	13344 (39.9)	3783 (35.7)	2533 (41.9)
Family history of CVD	30721 (44.8)	16879 (50.4)	5547 (52.4)	2129 (35.2)
Tobacco use				
Current smoker	13419 (19.6)	7474 (22.3)	2990 (2835)	1059 (17.5)
Former smoker	7134 (10.4)	4238 (12.7)	1392 (13.2)	583 (9.6)
Never smoke	48034 (70.0)	21771 (65.0)	6202 (58.6)	4410 (72.9)
Abdominal obesity	15307 (22.3)	9197 (27.5)	3237 (30.6)	1510 (25.0)
Alcohol use	21348 (31.1)	12151 (36.3)	4033 (38.1)	1293 (21.4)
Hypertension	28289 (41.3)	13034 (38.9)	4217 (39.8)	2737 (45.2)
Diabetes	2980 (4.3)	1603 (4.8)	554 (5.2)	301 (5.0)

[a] missing data

© 2021 Santosa A et al. *JAMA Network Open.*

eReference

1. Corsi DJ, Subramanian SV, Chow CK, McKee M, Chifamba J, Dagenais G, Diaz R, Iqbal R, Kelishadi R, Kruger A, Lanas F, López-Jaramilo P, Mony P, Mohan V, Avezum A, Oguz A, Rahman MO, Rosengren A, Szuba A, Li W, Yusoff K, Yusufali A, Rangarajan S, Teo K, Yusuf S. Prospective Urban Rural Epidemiology (PURE) study: Baseline characteristics of the household sample and comparative analyses with national data in 17 countries. Am Heart J 2013; 166:636-646.

© 2021 Santosa A et al. *JAMA Network Open.*
